# Eco-evolutionary Feedbacks from Non-target Species Influence Harvest Yield and Sustainability

**DOI:** 10.1038/s41598-018-24555-0

**Published:** 2018-04-23

**Authors:** Zachary T. Wood, Eric P. Palkovacs, Michael T. Kinnison

**Affiliations:** 1 School of Biology and Ecology, University of Maine, Orono, ME USA; 2 Ecology and Environmental Sciences Program, University of Maine, Orono, ME USA; 3 Ecology and Evolutionary Biology, University of California, Santa Cruz, CA USA

## Abstract

Evolution in harvested species has become a major concern for its potential to affect yield, sustainability, and recovery. However, the current singular focus on harvest-mediated evolution in target species overlooks the potential for evolution in non-target members of communities. Here we use an individual-based model to explore the scope and pattern of harvest-mediated evolution at non-target trophic levels and its potential feedbacks on abundance and yield of the harvested species. The model reveals an eco-evolutionary trophic cascade, in which harvest at top trophic levels drives evolution of greater defense or competitiveness at subsequently lower trophic levels, resulting in alternating feedbacks on the abundance and yield of the harvested species. The net abundance and yield effects of these feedbacks depends on the intensity of harvest and attributes of non-target species. Our results provide an impetus and framework to evaluate the role of non-target species evolution in determining fisheries yield and sustainability.

## Introduction

Harvest by humans is frequently associated with significant accumulating changes in the traits of targeted species^1,2^. In marine and freshwater systems, selective harvesting of the largest and oldest fish often favors smaller fish that mature earlier^3–6^, thereby driving trait changes that increase the fishing effort necessary to yield a consistent biomass haul^7,8^. Harvest-induced trait changes can also alter demographic parameters linked to sustainable stock growth or recovery of stocks following moratoria^9^. Concerns for these effects on sustainable yield and population abundance have prompted growing demand for evolutionary impact assessments^10,11^ that link different harvest intensities and patterns to anticipated dynamics of trait change, abundance, and yield over contemporary time scales (i.e., 50–100 years).

Recently, concerns for sustainability have further expanded to consider ways in which evolution in harvested species can cause cascading ecological changes in lower trophic levels^12–14^, sometimes reducing community stability and resilience^15^. To date, the common denominator to this work has been an almost singular attention to evolution of the harvested species themselves. In this study, we expand focus to ways in which harvested species abundance and yield could be impacted by evolution in lower trophic levels. We use an eco-evolutionary dynamic modelling framework^16–18^ to explore whether there is reason to cast our Darwinian net more broadly to consider trait changes and their ecological feedbacks in non-harvested members of communities.

Cascading community changes caused by harvest are likely to generate contemporary evolution in trophic levels below those occupied by harvested species. Many harvested species are at or near the apex trophic level in many communities^19^. Harvest-induced depression of these top trophic levels can therefore cause cascading density- and behaviorally-mediated changes in lower, non-harvested trophic levels^20,21^. Such changes in community structuring mirror changes in predation regimes that have generated many examples of contemporary evolution in lower trophic levels^22–24^. Indeed, contemporary evolution in response to changes in predation has been documented in all levels of pelagic food chains: piscivorous fishes^4^, planktivorous fishes^24–26^, zooplankton^27^, and phytoplankton^28,29^. This body of work not only suggests that cascading ecological changes due to harvest might drive evolution in lower trophic levels, but also hints at a trophic pathway by which such evolution could reciprocally feedback on the abundance dynamics of harvested species.

Although specific adaptations to predator or prey regimes are diverse, the nature of these adaptions can be broadly classified along a competition-defense tradeoff spectrum, in which feeding ability and vulnerability to predators are positively related. Such tradeoffs have been explored in a wide range of organisms, including fish^22,24^, plants^30^, insects^31,32^, algae^28,29,33^, and bacteria^34^. Competition-defense tradeoffs may be behavioral^22,35^, morphological^22,24^, physio-chemical^33,36^, or life-historical^37–40^. Importantly, a substantial body of theory and experimentation, much beginning with Pimentel’s^41^ pioneering work on the ‘genetic feed-back’, indicates that contemporary evolution along this tradeoff can substantially influence the abundance and stability of both predator and prey populations^28,29,42–44^. The population dynamical signatures of evolution along this trade-off have recently been uncovered in many “classic” predator-prey experiments, in which evolution was not originally considered^45^.

Here we investigated the eco-evolutionary consequences of contemporary evolution in non-harvested species during harvest. We sought to develop a generalized model to explore the scope (capacity) and pattern of evolution of non-target evolution and feedbacks on stability and yield of the harvested species, with the intent that our findings might serve to generate baseline predictions for future empirical and theoretical exploration. We took an eco-evolutionary dynamics approach, employing a multi-trophic-level, individual-based model^16,18^. Because other models exist to predict trait and ecological consequences of evolution in harvested species, we focus here on the evolutionary outcomes and feedbacks originating in non-harvested members of the community, which were modeled as discreet trophic levels. Genotypes and phenotypes of specific non-harvested trophic levels were allowed to evolve (eco-evolutionary model) or not (ecology-only model) in bifurcated model runs that split after harvest onset. These bifurcated runs allowed us to isolate eco-evolutionary from purely ecological processes. Although greater complexity might be added to our approach, the consistent and generalizable patterns we observed provide insight into specific conditions under which non-target evolution and eco-evolutionary feedbacks might be most overt and critical to harvest sustainability.

## Methods

### Data and code availability

The datasets generated and analyzed during the current study are available from the corresponding author on reasonable request. The computer code for this project is regularly updated for further research; the most recent version may be obtained from the corresponding author.

### Model overview

We used a generalized individual-based model to extend our eco-evolutionary framework beyond classic two-level predator-prey models to simulate a four trophic-level community in which the top or penultimate trophic levels (referred to as the top predator and secondary consumer, respectively) were subjected to a range of harvest intensities. We simulated evolution and ecology dynamically along a competition-defense tradeoff axis separately in each trophic level below the harvested level by allowing genotypes and phenotypes at that focal level to undergo selection based on the reciprocally interacting abundance dynamics of their own predators and prey. Evolution was an emergent property of this system^46^, with selection a byproduct of predator and prey dynamics and inheritance determined by genetic and environmental components. We analyzed the effect of contemporary evolution in non-harvested species on abundance and yield of the harvested species by comparing bifurcated models: an *eco-evolutionary model*, in which evolution was allowed to continue after the initiation of harvest, and an *ecology-only model*, in which genotypes were frozen at the harvest onset mean, although environmental variation was retained. This approach allowed us to compare models to determine the extent to which evolution in non-harvested species impacts yield and sustainability of harvest for targeted species.

#### Competition-defense tradeoffs

Preliminarily, we tested a wide range of competition-defense tradeoff slopes (Fig. S1) in trophic levels below the harvested species to determine the trait space within which evolution in response to harvest would occur. We calculated tradeoff slope as:

1 $$S=\frac{\,\frac{\partial a}{\partial G}\,}{\,\frac{\partial v}{\partial G}\,}$$

In which *S* = tradeoff slope, *a* = attack rate on resources, *v* = vulnerability to predators, and *G* = genotype (coded as a continuous quantitative trait). We ran simulations at moderate harvest levels (levels that would visibly reduce the abundance of the harvested species but would almost never lead the harvested species to extirpation). For these initial runs we simulated evolution separately for each trophic level (i.e. we allowed only one trophic level to evolve at a time). For all tested trophic levels in all model structures, we observed a central range of competition-defense tradeoff slopes that led to marked evolution after harvest onset (see Results and Discussion). Because our primary goal was to discern generalized patterns of potential feedbacks from non-target evolution (to inform future data collection and investigations), we selected competition-defense tradeoff ratios roughly in the middle of these slope ranges for the following analyses.

#### Single trophic level evolution

Using the above selected values for competition-defense tradeoff ratios, we simulated a range of consistent effort harvest from negligible harvest to overharvest resulting in harvested species extirpation. We ran a separate model for each non-harvested trophic level, allowing only that trophic level to evolve. We observed differences in eco-evolutionary and eco-only model results for harvested species abundance, yield, and stability across the harvest intensity gradient. We attributed differences between these two models to eco-evolutionary processes rooted in evolution in the lone evolving trophic level.

#### Multi-trophic evolution

We subsequently tested for effects of evolution in multiple non-harvested trophic levels on the harvested species. Using our four-trophic level model in which the top predator was harvested, we allowed pairs of trophic levels to evolve to examine the potential for evolutionary reinforcement or compensation at multiple trophic levels. We also ran models in which all trophic levels below the harvested species evolved and varied food web length (four or three trophic levels total) to examine how “fishing down the food web”^19^ would change the net effect of evolution on the harvested species.

### Model design and details

**Figure 1:**
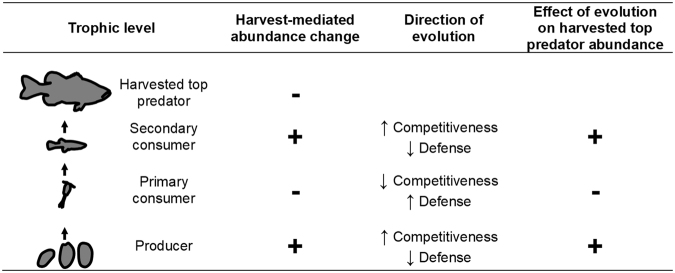
Harvest-induced eco-evolutionary trophic cascades. Cascading harvest-mediated abundance changes cause evolution in lower, non-target species, which then feeds-back to bolster or undermine the harvested top predator. Directions of abundance changes, evolution, and feedbacks alternate predictably down the food chain. Results are from an individual-based model with four trophic levels, each feeding exclusively on the level below it. Patterns are robust to models in which the penultimate trophic level (secondary consumer) was harvested instead of the top predator.

We built an individual-based model framework using Matlab R2015b software. The model uses iterative Monte Carlo methods to simulate four discrete populations, with each population constituting an entire trophic level (Fig. 1).

#### Representing populations, genotypes, and phenotypes

Each population was represented by a data table, in which each row represented an individual and each column represented a trait. Births were appended to each table; deaths were deleted.

Each individual in each population had a quantitative trait genotype influencing its competition-defense phenotype. Our assumption of polygenic inheritance is grounded in the premise that such performance phenotypes likely reflect the additive influence of many interacting traits that may or may not be polygenic themselves. For efficiency and generalizability, we produced subsequent generations by sampling directly from standing genetic variation (i.e. asexually) and applying a mutational component. Sampling to produce offspring was weighted by the survival and reproductive probabilities of current organisms. Such inheritance is generalizable as an additive polygenic trait^47^, and is robust to most dominance and recombination structures, while avoiding variance deflation^48^. We allowed a small chance of mutational input to variation during each reproductive event:

2 $${G}_{offspring}={G}_{parent}+Binomial\,({P}_{mutation})\ast Normal\,(0,{\sigma }_{mutation})$$

*G* = genotype; *P*_*mutation*_ = mutation probability; and *σ*_*mutation*_ = average mutation severity.

Mutation probability was set to 0.12 for all trophic levels, consistent with mutational inputs for a highly polygenic phenotype (i.e. mutation is rare^49–51^, but an effectively large number of loci makes mutations likely). The standard deviation of mutation severity was set to 0.05 for all trophic levels. The starting mean and standard deviation for *G* were 0 and 0.25, respectively. In our model, the individual’s genotype simultaneously defines its competitiveness and defense, defined proximately as its attack rate, vulnerability, and death rate. Therefore, these three characteristics covaried, consistent with observed competition-defense tradeoffs (see Introduction). Finally, consistent with quantitative polygenic inheritance, attack rate, vulnerability, and death rate were all affected by a coefficient of environmental variation error term (noise), with the net effect that effective heritability was roughly 0.6.

3 $${a}_{i}=({a}_{0}+{\epsilon })(100+A{G}_{i})$$

4 $${v}_{i}=({v}_{0}+{\epsilon })(100+V{G}_{i})$$

5 $${d}_{i}=({d}_{0}+{\epsilon })(100+M{G}_{i}^{2})$$

*a*_*i*_ = attack rate; *a*_0_ = inherent attack rate; *ϵ* = normally distributed error term; *A* = contribution of focal gene to attack rate; *G*_*i*_ = genotype; *v*_*i*_ = vulnerability; *v*_0_ = inherent vulnerability; *V* = contribution of focal gene to vulnerability; *d*_*i*_ = death rate; *d*_0_ = inherent death rate; *M* = contribution of focal gene to death rate.

A heritability of 0.6 is relatively high^52^, but we suggest this is justifiable on the grounds that contemporary evolution of performance tradeoffs leverages heritability of multiple traits, including component traits with higher than average heritability (but see Discussion).

We manipulated the tradeoff ratio (*A*/*V*) to determine the slope of the competition (*A*) – defense (*V*) tradeoff for each population. A high tradeoff ratio indicated “cheap” competition, while a low tradeoff ratio indicated “cheap” defense (Fig. S1). Death rates increased quadratically with extreme phenotypes in order to prevent runaway evolution in cases of complete predator absence and ensure some measure of canalization^43^.

#### Iterating events

Births were dependent on the total number of prey consumed by each individual, which were in turn dependent on individual phenotypic attack rate and the pool of available prey (determined by the death rate of the next lower trophic level):

6 $${L}_{i}={b}_{i}\frac{\frac{{a}_{i}}{1+{h}_{i}{a}_{i}{N}_{prey}}{N}_{preyeaten}}{\overline{(\frac{a}{1+ha{N}_{prey}})}N}$$

*L*_*i*_ = birth probability; *b*_i_ = conversion efficiency; *a*_*i*_ = attack rate; *h*_*i*_ = handling time; *N*_*prey*_ = prey abundance; *N*_*prey eaten*_ = number of prey whose deaths were from predation (see death formula).; *N* = number of consumers. Note that the denominator is a mean quantity for the entire population.

We determined deaths through an intrinsic mortality rate plus deaths from predation, as determined by an individuals’ phenotypic vulnerability to predators:

7 $${d}_{{P}_{i}}=\overline{(\frac{{a}_{pred}}{1+{h}_{pred}{a}_{pred}N})}{v}_{i}{N}_{pred}$$

${d}_{{P}_{i}}$ = death probability from predation; *a*_*pred*_ = predator attack rate; *h*_*pred*_ = predator handling time; *N* = number of consumers; *N*_*pred*_ = number of predators; *v*_*i*_ = vulnerability to predators.

8 $${D}_{i}=1-(1-{d}_{i})(1-{d}_{{P}_{i}})$$

*D*_*i*_ = total death probability; *d*_*i*_ = intrinsic death probability; ${d}_{{P}_{i}}$ = death probability from predation.

We accounted actual births by sampling a Poisson distribution with a mean of *L*_*i*_. We accounted actual deaths by sampling a binomial distribution with a mean of *D*_*i*_. To quantify the number of deaths actually from predation, we summed the proportion of the death probability that was due to predation for all dead individuals:

9 $${N}_{eaten}=\sum _{i}(\frac{{d}_{{P}_{i}}}{{D}_{i}}|dead)$$

*N*_*eaten*_ = number of deaths from predation; ${d}_{{P}_{i}}$ = death probability from predation; *D*_*i*_ = total death probability.

We used this term to inform the number of births at the next higher trophic level (see Eqn. 6). Producers consumed a finite but replenishing resource.

#### Adding harvest

We assumed a consistent effort harvest with a small normally-distributed (5%) error term. We added harvest by modifying the death rate of the top or penultimate predator:

10 $${D}_{i}=1-(1-{d}_{i})(1-{d}_{{P}_{i}})(1-f-{{\epsilon }}_{f})$$

*D*_*i*_ = death rate of the harvested species; ${d}_{{P}_{i}}$ = death rate from predation sources; *d*_*i*_ = inherent death rate; *f* = harvest intensity; *ϵ*_*f*_ = harvest error from variability, sampled from a normal distribution.

We calculated yield as the proportion of deaths each iteration that were due to harvest:

11 $$Y=\sum _{i}(\frac{f+{{\epsilon }}_{f}}{{D}_{i}}|dead)$$

*Y* = harvest yield; *f* = harvest intensity; *ϵ*_*f*_ = harvest error; *D*_*i*_ = total death rate of the harvested species.

We tested a range of harvest intensities from harvests that had no appreciable impact on the harvested species to overharvests that led to harvested species collapse.

#### Parameterizing the model

We used parameter combinations that generated a primary producer population size that was computationally manageable. We then used geometric changes in attack rate, handling time, and death rate to create a community in which population size decreased and generation time increased with increasing trophic level (Table 1). Attack rates, vulnerability, and death rates were modified by genotype in focal, evolving trophic levels (see *Representing populations, genotypes, and phenotypes*); the values in Table 1 represent the starting values that were then modified by genotypes.

**Table 1:** Starting population parameter values for individual-based model simulations.

Symbol	Parameter	Producer value	Geometric change with increasing trophic level
a_0_	Attack rate	6 ∗ 10^−6^	/ 2
v_0_	Vulnerability	1	^*^ 1
b	Conversion efficiency	1 ∗ 10^−3^	^*^ 1
h	Handling time	2 ∗ 10^−10^	^*^ 100
d_0_	Death rate	1 ∗ 10^−3^	/ 4

#### Running the model

**Figure 2:**
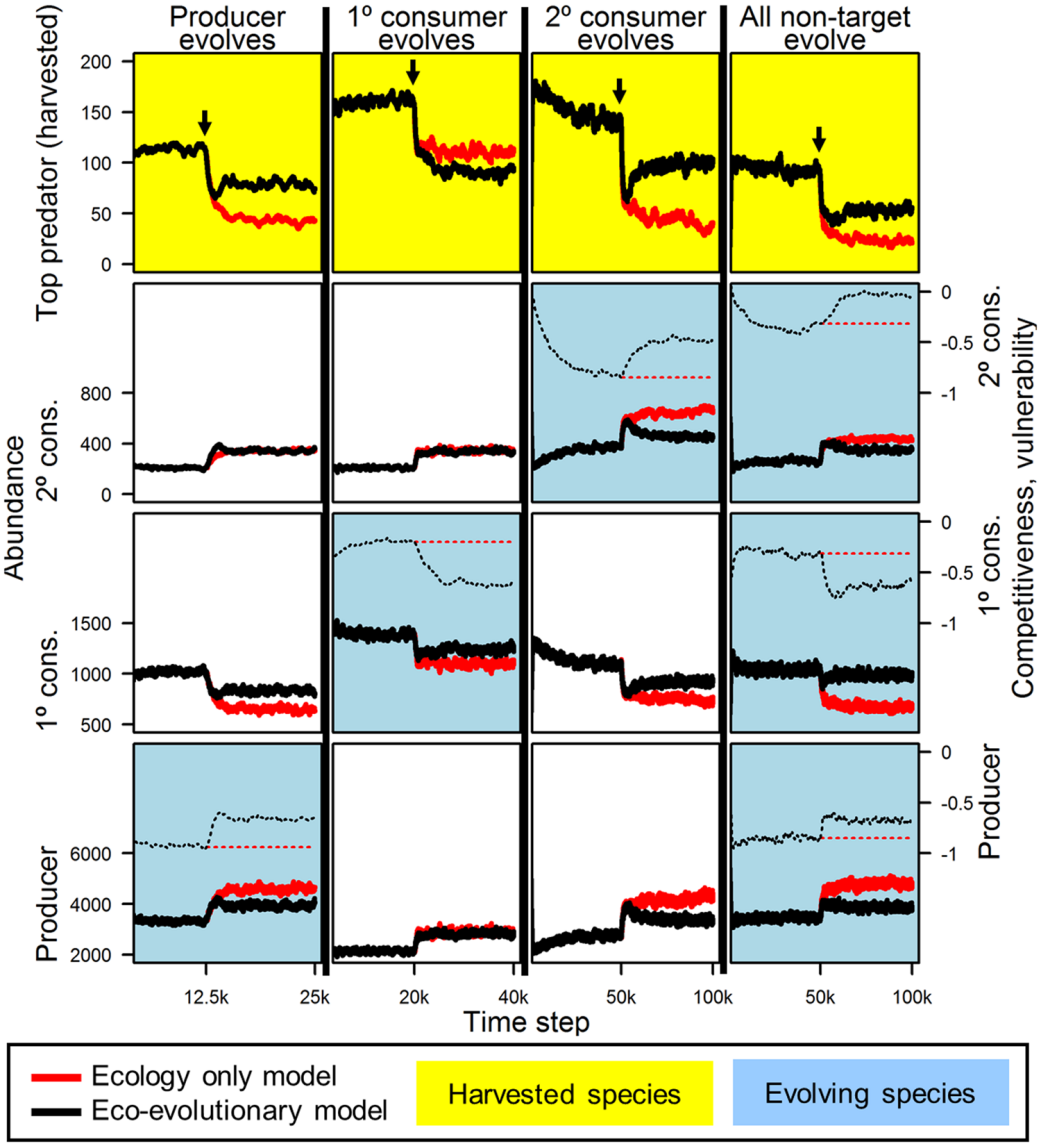
Eco-evolutionary consequences of non-target species evolution during harvest. Harvest-mediated evolution in non-target trophic levels feeds-back to undermine or bolster the harvested top predator. Each column represents a unique set of simulations. Odd-numbered trophic levels below the harvested species evolve increased competitive ability and decreased defense (increased vulnerability) during harvest, which bolsters the harvested species; even numbered trophic levels evolve decreased competitive ability and increased defense (decreased vulnerability), undermining the harvested species. Black lines show average results from 12 simulations with evolution on (eco-evolutionary models); red lines show average results from 12 simulations in which trait values were fixed at pre-harvest means (ecology only models). Thick lines show population abundances; thin dashed lines show genotype means, which code for competitiveness and vulnerability to predators. Harvest of top predator begins at arrows. Competition-defense tradeoff ratios were set to maximize evolutionary potential (Fig. 3). See Fig. 1 for model structure schematic.

We ran about 200 batches of simulations for each trophic level below the harvested species. In each batch, we allowed only one, non-harvested trophic level to evolve (Fig. 2). We initially ran the model for 12,500 iterations (when the lowest trophic level evolved; 20,000 when the next higher trophic level evolved, and 50,000 when the penultimate trophic level evolved) to attain quasi-equilibrium. We then initiated harvest and ran for an additional 12,500 iterations under harvest conditions (when the lowest trophic level evolved; 20,000 when the next higher trophic level evolved, and 50,000 when the penultimate trophic level evolved). In this model each iteration represents a finite time step. While the total number of time steps is very large, the effective generation time (*T*) of each trophic level spans 1.5 to 640 iterations. Using the definition of a generation as the mean time between the birth of an individual and the birth of its offspring, a 10,000 iteration time window in our model equated with 16 generations of the top predator (*T* = 639.50), 185 generations of the secondary consumer (*T* = 54.15), 580 generations of the primary consumer (*T* = 17.19), and 6450 generations of the producer (*T* = 1.55).

**Figure 3:**
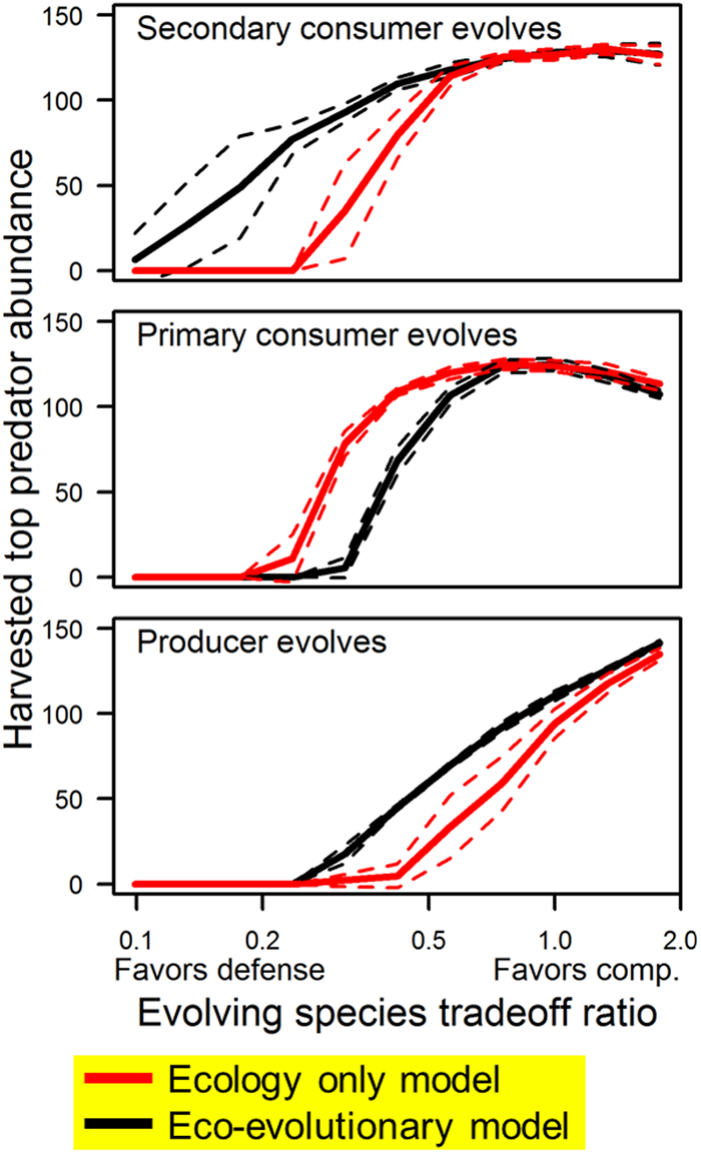
Competition-defense tradeoffs and eco-evolutionary potential. Competition-defense tradeoff ratio in non-target species influences the difference between ecological (red) and eco-evolutionary (black) model predictions of harvest-species abundance. “Cheap defense” tradeoffs (left side) lead to system destabilization prior to harvest. “Cheap competition” tradeoffs (right side) lead to similar predictions from both models. Intermediate competition-defense tradeoffs lead to harvest-induced evolution in non-target species (Fig. 2), which feeds-back to bolster or undermine the harvested top predator. Solid and dashed lines indicate mean ± one standard deviation for 12 model runs at each tradeoff ratio value. See Fig. 1 for model structure schematic.

**Figure 4:**
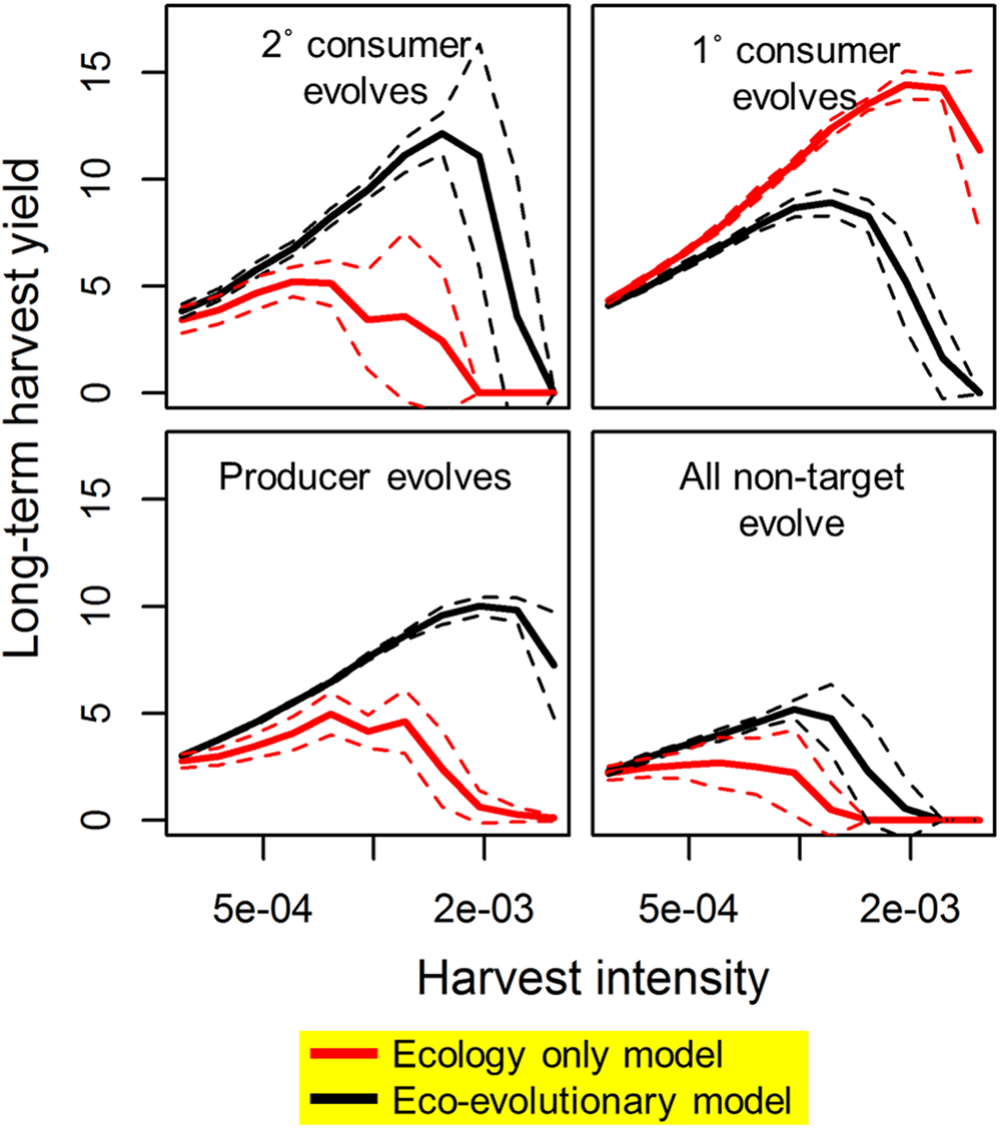
Harvest yield predictions from ecology only versus eco-evolutionary models. Evolution in non-target trophic levels can lead to significantly higher or lower long-term harvest yield, maximum sustainable yield, and appropriate harvest intensity. Considering ecological (red) processes only and neglecting eco-evolutionary (black) model predictions can lead to top predator overharvest, extirpation, or foregone yield, depending on which trophic levels evolve. Dashed lines show ± one standard deviation for 12 model runs. See Fig. 1 for model structure schematic.

At initiation of harvest, we split the model into two parallel models: one in which evolution continued and one in which genotypes of the evolving population were frozen at pre-harvest means. The former model therefore includes eco-evolutionary effects of harvest, while the latter includes only ecological effects. We ran these simulations with harvest of either the top or penultimate trophic level (referred to as the top predator and secondary consumer, respectively). To determine the range of harvest intensities to explore with our model, we ran the models with no evolution occurring at a wide range of harvest intensities and selected an initial harvest intensity that produced a modest depression in the abundance of the harvested species (*f* = 0.0009 for the top predator; 0.0077 for the secondary consumer). Using this harvest intensity as a setpoint, we ran simulations across a wide range of competition-defense tradeoff ratios (Figs 3, S-7) to examine the sensitivity of harvest stability and yield to trade-off ratio. We then subsequently selected a setpoint tradeoff ratio at each trophic level that generated strong differences between eco-only and eco-evolutionary models and assessed the sensitivity of evolution and feedbacks to varying harvest strengths (Figs 4, S-8). For the simulations involving *top predator harvest* (and fishing down the food web example) our setpoint ratios (*A*, *V*) were (19.1, 52.5),(22.0, 45.4), (29.5, 39.3) for the secondary consumer, primary consumer, and producer, respectively. For simulations involving *secondary consumer harvest*, the setpoint ratios (*A*, *V*) were (17.0, 58.8), (20.5, 48.8) for the primary consumer and producer, respectively. For all models, canalization parameters (*M*, see *Representing populations, genotypes and phenotypes*) were set to 25.0 for evolving trophic levels.

## Results and Discussion

Comparing bifurcated simulations consistently revealed a harvest-driven eco-evolutionary trophic cascade, in which upper trophic level harvest led to dynamically coupled evolutionary and ecological changes at proximate and distant trophic levels (Figs 1 and 2).

### Harvest induces an eco-evolutionary trophic cascade

For any given trophic level, we found that evolution followed a general pattern. Odd-numbered trophic levels (starting with the level below harvested) evolved increased competitive ability and lower defense, and even-numbered trophic levels evolved greater defense and lower competitive ability (Figs 1 and 2). This flip-flopping pattern is analogous to the common depiction of alternating dominance of predation and competition as regulators of abundance in classical density-mediated trophic cascades^53^. Notably, selection at each intermediate trophic level was mutually reinforced by the relative abundances of its predators and prey, providing particularly strong and consistent selection along the competition-defense tradeoff. An abundant trophic level with relatively few predators and prey faces weaker selection for defense and stronger selection for competition (e.g. secondary consumers, Fig. 1), whereas a less abundant trophic level with numerous predators and prey faces stronger selection for defense and weaker selection for competitiveness (e.g. primary consumers, Fig. 1). In this respect, abundance and competitiveness tended to track together (Fig. 2). Shortening of the food chain to three trophic levels and harvesting the secondary consumer (“fishing down the food web”)^19^ led to evolutionary reversal in subsequent non-target trophic levels (Fig. S4), suggesting that the most important factor in determining the direction of evolution in any trophic level was that level’s food web distance from the harvested species.

Importantly, our model shows the direction of causation was not exclusively one of ecology driving evolution; evolution also fed back to affect ecology. For example, adaptive evolution of more competitive but vulnerable prey of a harvested predator tended to feed back positively on that predator’s abundance, causing an increase in the population size of the harvested predator. These results are consistent with studies of antipredator evolution and predator abundance in simpler predator-prey systems^28,34,54–56^.

### Evolution in lower trophic levels feeds-back to bolster or undermine harvested species

Eco-evolutionary feedbacks again followed a consistent pattern that transmitted in a bottom-up fashion to the harvested species. Evolution in odd-numbered trophic levels (again starting with the level below harvested) fed back positively on abundances of the harvested species, though the net extent of the population increase depended on reinforcing or opposing evolution at other trophic levels (Figs 2, 4, S3). Evolution in even-numbered trophic levels fed back negatively on the abundance of the harvested species. Simultaneous evolution at multiple trophic levels resulted in stronger or weaker net demographic effects on the harvested species, depending on which levels evolved (Figs 2, S3). Neighboring trophic levels’ eco-evolutionary feedbacks tended to cancel (a form of cryptic eco-evolutionary dynamics)^57^ while once-removed trophic levels’ eco-evolutionary feedbacks were reinforcing (Fig. S3).

These eco-evolutionary feedbacks were able to significantly affect yield and sustainability in our simulated harvest fishery (Fig. 4). Evolution of increased competition in odd-numbered trophic levels below the harvested species increased yield and sustainability. Evolution of increased defense in even-numbered trophic levels decreased yield, and lowered the harvest rates associated with fisheries collapse (Fig. 4). Evolutionary effects on yield and abundance (i.e. differences between eco-evolutionary and ecology only models) were most pronounced at high harvest levels, where evolution of non-target species was at times the difference between obtaining the highest possible yields and complete harvest collapse (Fig. 4). Results were similar when the penultimate trophic level (secondary consumer) was harvested (Figs S5–S7), although abundance changes in the harvested species were lesser in magnitude.

### Competition-defense tradeoff slope dictates eco-evolutionary potential

At all trophic levels, evolution and its ecological feedbacks were most pronounced at intermediate to somewhat defense-biased tradeoffs, where fitness gains or losses from defense were accompanied by similar or slightly smaller changes in competitiveness (Figs 3, S7). Tradeoffs that favored very cheap defense (large changes in defense come with small changes in competitiveness) resulted in community destabilization and extinctions before harvest had started, whereas tradeoffs that favored cheap competition (large change in competitiveness associated with small changes in defense) led to little evolutionary change in response to altered predation regimes. The competition-defense tradeoff bias that led to the greatest difference between eco-evolutionary and ecology only models became more defense-biased with increasing trophic level (Figs 3, S7). These findings are consistent with experiments showing stronger eco-evolutionary feedbacks from balanced than unbalanced tradeoffs in a simpler predator-prey system^28^.

### Model expansions and future work

Our models reveal consistent patterns that should inform future study in real-world ecosystems. Future work for specific systems should consider nuances and complexities that fall within three broad categories:

#### Factors affecting the pace of evolution

Factors affecting the relative pace of evolution at different trophic levels (e.g. genetic variation, generation time, heritability), should not necessarily change the scope of the patterns we observed here, but will change the timescales on which patterns could be observed. Lower trophic levels in particular, by virtue of their larger population sizes and often shorter generation times, could evolve more quickly in the initial face of harvest on higher trophic levels and thus dominate in early harvest-driven feedbacks. We ran our models with simple assumptions about the relative generation times (see Methods), and long enough to accommodate quasi-equilibrium evolution in any trophic level, but a more nuanced understanding of relative evolutionary rates and transitory dynamics is likely important for detecting patterns and understanding specific outcomes on the management timeframes of real world harvest systems.

#### Factors affecting the scope of evolution

Eco-evolutionary dynamics in our models are strongly nuanced by the form of the competition-defense tradeoff ratios (Fig. 3). While there is strong theoretical and empirical basis for this tradeoff across diverse species and ecosystems (see Introduction), it remains poorly characterized for many species in harvested ecosystems. Because the goal of this study was to assess the scope for evolution and its feedbacks, our models centered on tradeoff-ratios for which evolution was likely to happen, but it should be noted that there exist areas of parameter space for which the eco-evolutionary and ecological expectations were relatively similar (Fig. 3). This result suggests that future research should place a priority on quantifying the form and slope of these tradeoff ratios for species in harvested ecosystems.

The underlying mechanisms for competition-defense tradeoffs may be both shared and unique across trophic levels. Certain changes in size or life history may be common tradeoff responses at multiple trophic levels^30,36,37,39^. Other responses may be more unique, such as greater reliance of primary producers on investments in defense compounds that reduce growth rate^29,33,34,58^, and greater reliance in upper trophic levels on complex behavioral competition-defense tradeoffs^22,35^. Although we would contend that competition-defense tradeoffs are broadly universal, it remains to be empirically determined whether and how specific tradeoff mechanisms might modify the scope for eco-evolutionary feedbacks, as might occur if some of these mechanisms are associated with very different tradeoff slopes.

Plastic, rather than genetic, trait change along a competition-defense tradeoff axis could lead to similar patterns to those observed in this model, and should not be ignored when considering real-world systems. Furthermore, although phenotypic plasticity may modify the strength of the genetic evolutionary response to harvest^59,60^, plastic responses to harvest-induced trophic cascades may be at least as ecologically impactful as genetic responses^21,61^, and likely occur on more immediate time scales.

We modelled a necessarily simplified food chain with discrete evolving trophic levels. The broad phenotypic and feedback patterns we describe are likely robust to the number of evolving species at a given trophic level so long as there is genetic variation (within or among species) along a competition-defense tradeoff. Indeed, the typically greater phenotypic and genetic variation among species could provide faster rates of net phenotypic change. In complex food webs, cascading responses are likely to involve change in both species and intraspecific diversity^53,62^.

#### Factors affecting the scope of eco-evolutionary feedbacks

Ecosystems vary in their connectivity and interspecific interaction strengths, and this variation can dictate the potential for cascading ecological impacts and system stability^63–65^. For example, omnivory, in which one organisms consumes individuals from multiple adjacent trophic levels, might be predicted to alter the strength of the eco-evolutionary dynamics we described here, either by dampening the net feedback—if omnivores integrate opposing feedbacks at neighboring trophic levels—or strengthening the net feedback—if large changes in omnivore abundance generate selection in the same direction at multiple adjacent trophic levels below the omnivore. Along these lines, we would suggest that ecological metrics of cascade strength^66,67^ or interaction strength^68,69^ might be used as an initial means to identify harvested communities where strong non-target evolution and eco-evolutionary feedbacks may be most evident and influential.

## Conclusions

The capacity for large differences between purely ecological versus eco-evolutionary models (Figs 2–4) in our study supports many prior calls for greater consideration of evolutionary processes when managing biological resources^17,70–72^. Under some scenarios, the predictions from ecology-only simulations differed substantially from eco-evolutionary simulations, suggesting that failure to account for evolution in non-target species could appreciably influence risks associated with overharvest or foregone yield (Fig. 4). Likewise, our results hint that harvesting at some trophic levels might be more prone to positive or negative feedbacks than others due to compensatory effects at other trophic levels. Taken broadly, our findings suggest that evolutionary impact assessments that focus exclusively on evolution in harvested species may provide an incomplete picture of evolution’s role in harvested ecosystems. However, this result does not imply that evolutionary management is complex beyond reach, or that non-target evolution is intrinsically bad for fisheries outcomes. While our model highlights some considerations that may ostensibly complicate real-world resource management, it also highlights general patterns that are testable in natural systems and processes that could convey a degree of resiliency in harvested ecosystems. Finally, our results imply a logical next step of investigating the interaction between evolutionary processes in harvested trophic levels (i.e. harvest-induced evolution) and evolutionary processes in lower trophic levels.

## Electronic supplementary material

Supplement
