# Supplementary material for: Eco-evolutionary Feedbacks from Non-target Species Influence Harvest Yield and Sustainability
Source: Sci Rep. 2018 Apr 23;8:6389. doi: 10.1038/s41598-018-24555-0 (PMC5913267; doi:10.1038/s41598-018-24555-0)
Supplement: Supplementary file 1 — Supplement [file 41598_2018_24555_MOESM1_ESM.pdf]

SUPPLEMENT

ECO-EVOLUTIONARY FEEDBACKS FROM NON-TARGET SPECIES INFLUENCE HARVEST YIELD AND SUSTAINABILITY

In revision for *Scientific Reports*

Zachary T. Wood,[1,2]* Eric P. Palkovacs,[3] Michael T. Kinnison[1]

[1]School of Biology and Ecology, University of Maine, Orono, ME, USA.

[2]Ecology and Environmental Sciences Program, University of Maine, Orono, ME, USA.

[3]Ecology and Evolutionary Biology, University of California, Santa Cruz, CA, USA

*Correspondence to: zachary.t.wood@maine.edu

Verbal explanations of model equations

(2) Offspring genotype = parent genotype + mutation severity if mutation happens

(3,4,5) Attack rate, etc = inherent attack rate + environmental noise * effect of focal gene

(6) Birth probability = conversion efficiency * individual consumption rate / average consumption rate * total number of prey consumed / number of consumers. Consumption follows a type II functional response.

(7) Death probability from predation = predator consumption rate * vulnerability to predators * number of predators

(8) Total death probability = 1 – probability of surviving all hazards

(9) Number of prey eaten = fraction of death probability from predation * number of dead individuals

(10) Total death probability = 1 – probability of surviving all hazards (now including fishing)

(11) Number of fish caught = fraction of death probability from fishing * number of dead individuals

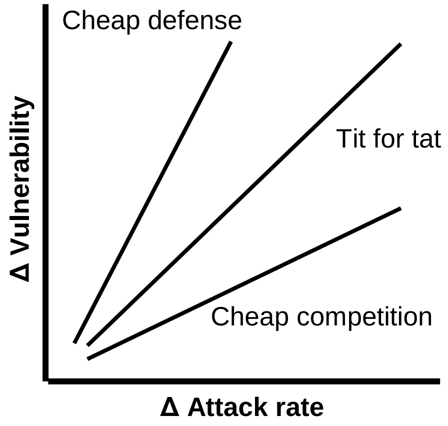

Figure S1. Competition-defense tradeoffs. Competition – defense tradeoffs in our model were realized by modifying the degree to which attack rate and vulnerability increased with increasing genotype values. “Cheap competition” occurs when changes in genotype can increase attack rate with little increase in vulnerability, and vice-versa.

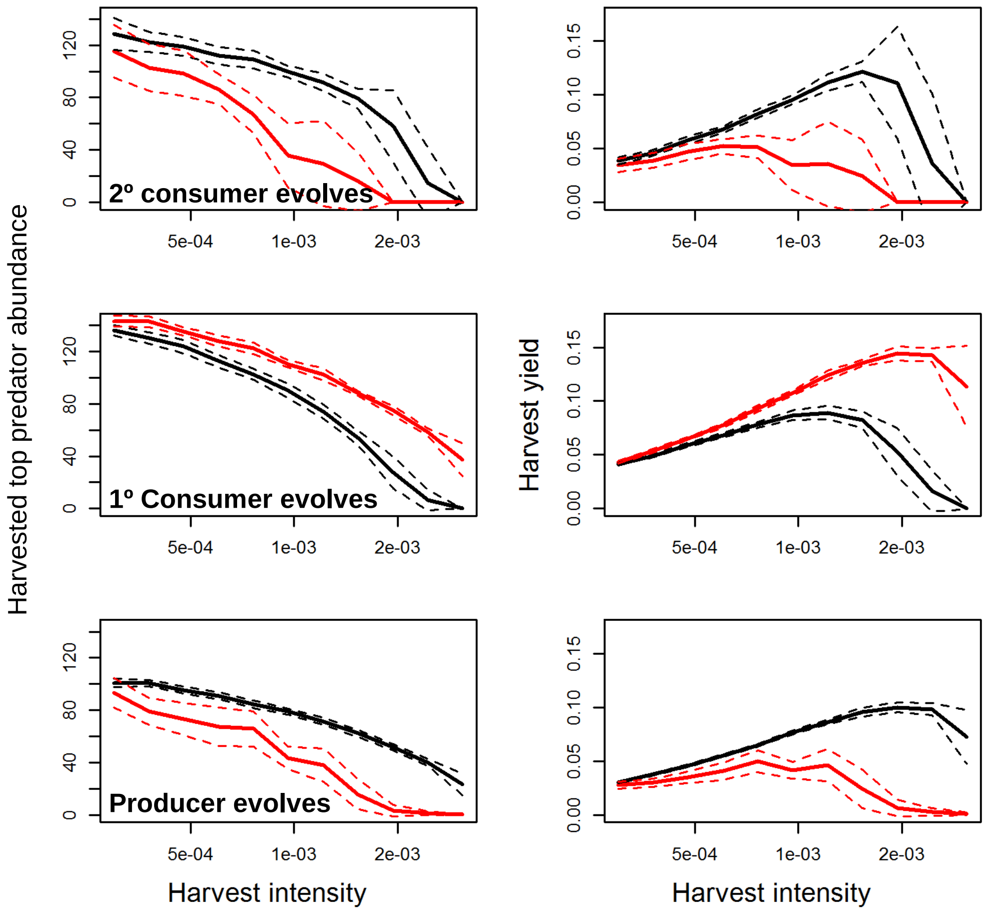

Figure S2. Harvest yield and sustainability when lower, non-target trophic levels evolve. Evolution in trophic levels below the harvested top predator alternately dampened and exacerbated harvest effects with decreasing trophic level. **Black** lines represent outcomes with evolutionary and ecological processes included; **red** lines represent outcomes with evolution frozen and only ecological processes following the initiation of harvest. Evolution in odd-numbered trophic levels increased harvested species yield and stability, while evolution in even-numbered trophic levels decreased yield and stability. Lines represent mean ± one standard deviation for twelve runs per each point

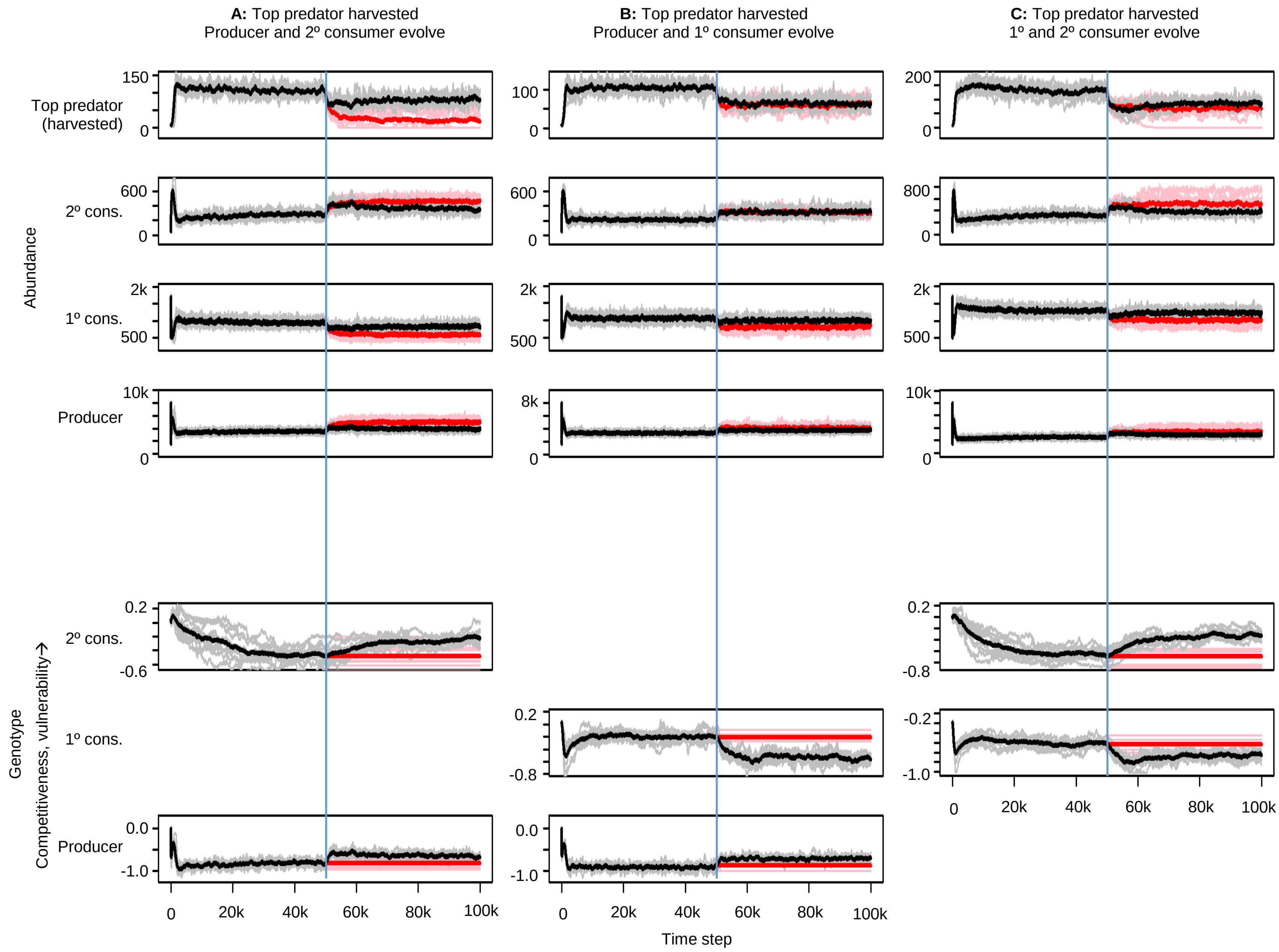

A: Top predator harvested
Producer and 2º consumer evolve
B: Top predator harvested
Producer and 1º consumer evolve
C: Top predator harvested
1º and 2º consumer evolve
Top predator (harvested)
2º cons.
1º cons.
Producer
Abundance
Genotype
Competitiveness, vulnerability→
Time step
0
20k
40k
60k
80k
100k

Figure S3. Multi-trophic non-target evolution during harvest of a top predator. Once-removed trophic levels (panel A) evolved in similar directions to create a large combined bolstering effect on the harvested species. Neighboring trophic levels (panels B and C) evolved in opposite directions, with effects on the harvested species roughly cancelling. **Black** lines show the average (dark line) and 12 example runs (gray lines) when evolution was allowed to proceed. **Red** lines show the average (dark line) and 12 example runs (pink lines) when evolution was frozen at harvest onset (vertical **blue** lines).

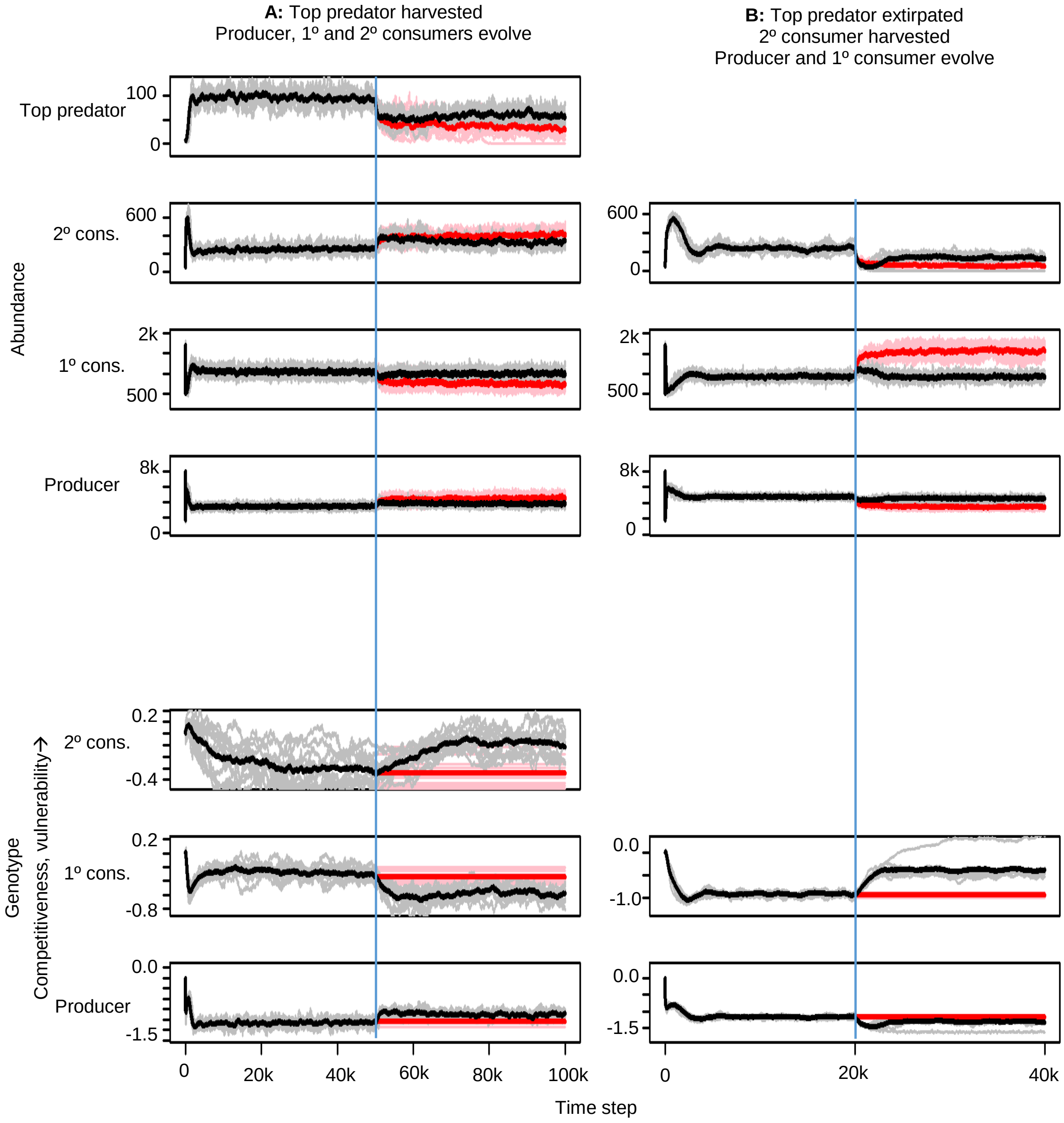
A: Top predator harvested
Producer, 1º and 2º consumers evolve
B: Top predator extirpated
2º consumer harvested
Producer and 1º consumer evolve
Abundance
Top predator
100
0
2º cons.
600
0
1º cons.
2k
500
Producer
8k
0
Genotype
Competitiveness, vulnerability→
2º cons.
0.2
-0.4
1º cons.
0.2
-0.8
0.0
-1.0
Producer
0.0
-1.5
0
20k
40k
60k
80k
100k
Time step

Figure S4. Effects of fishing down the food web. Shortening the food chain from 4 (panel A) to 3 trophic levels (panel B) and harvesting secondary consumers (now the top tropic level) led to evolutionary reversal in lower trophic levels, though the net effect of evolution was to bolster the harvested species in both scenarios. **Black** lines show the average (dark line) and 12 example runs (gray lines) when evolution was allowed to proceed. **Red** lines show the average (dark line) and 12 example runs (pink lines) when evolution was frozen at harvest onset (vertical **blue** lines).

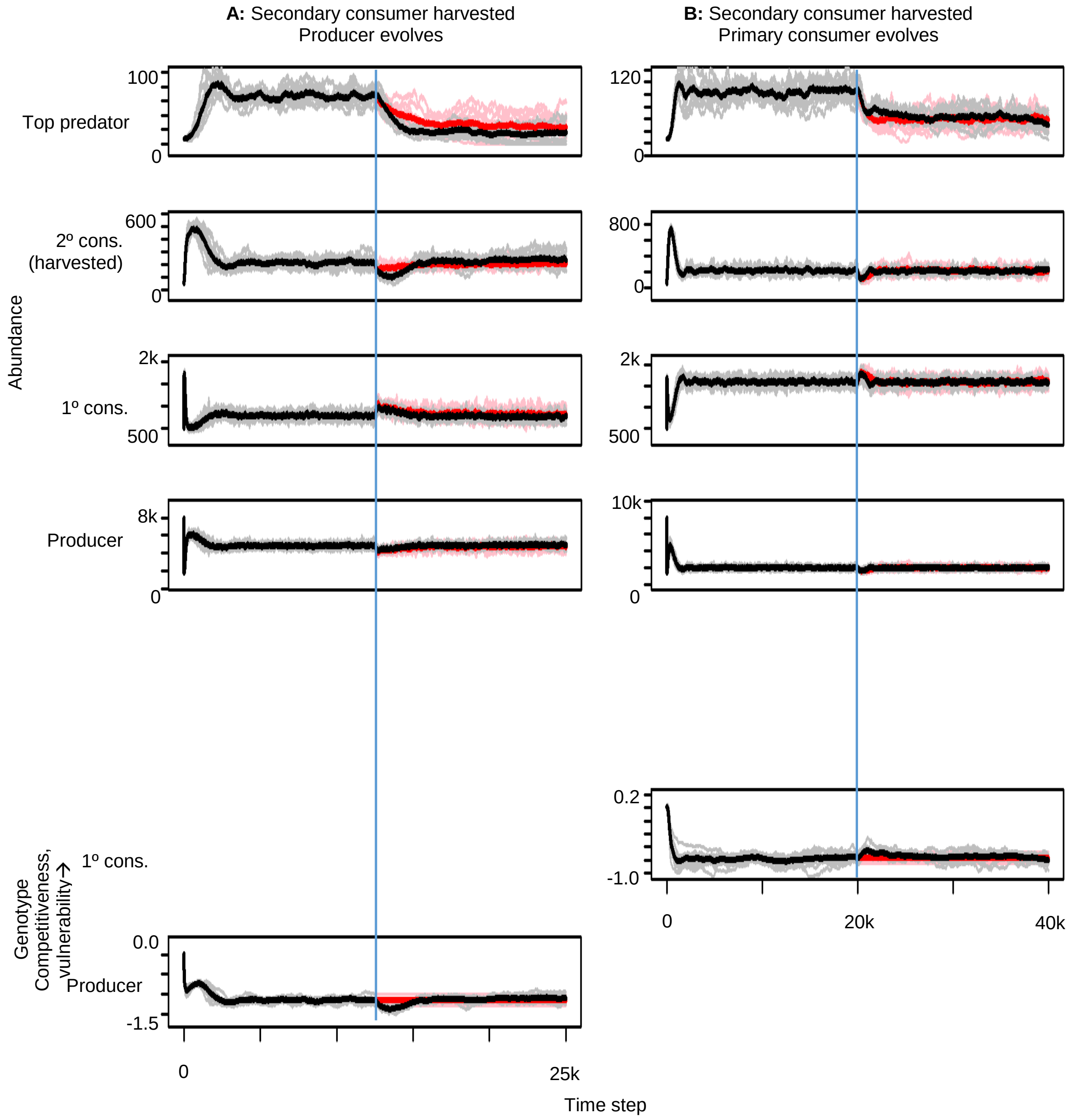
A: Secondary consumer harvested
Producer evolves
B: Secondary consumer harvested
Primary consumer evolves
Top predator
2º cons.
(harvested)
1º cons.
Producer
Abundance
Genotype
Competitiveness,
vulnerability→
100
0
600
0
2k
500
8k
0
0.0
-1.5
120
0
800
0
2k
500
10k
0
0.2
-1.0
0
25k
0
20k
40k
Time step

Figure S5. Eco-evolutionary consequences of non-target species evolution during harvest of the secondary consumer (penultimate trophic level). Even-numbered trophic levels below the harvested species (panel A) evolved increased defense and weakly decreased the abundance of the top predator; odd-numbered trophic levels (panel B) evolved increased competitive ability and lead to a weak bolstering of the top predator. **Black** lines show the average (dark line) and 12 example runs (gray lines) when evolution was allowed to proceed. **Red** lines show the average (dark line) and 12 example runs (pink lines) when evolution was frozen at harvest onset (vertical **blue** lines).

Figure S6.

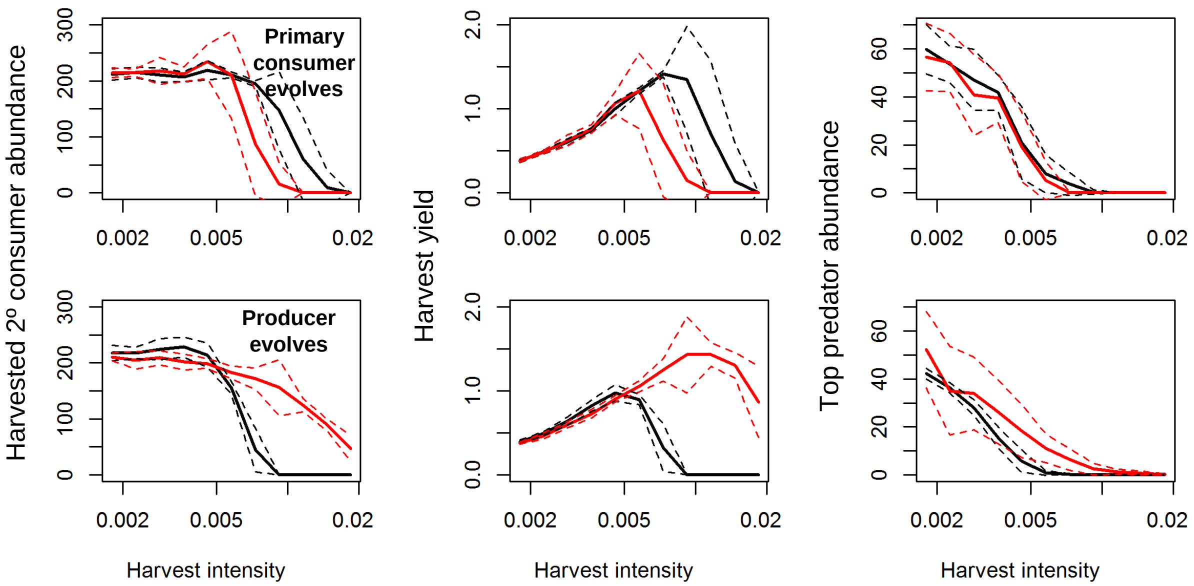

Harvest yield and sustainability when lower, non-target trophic levels evolve. Secondary consumer (penultimate trophic level) harvested. Evolution in trophic levels below the harvested species alternately dampened and exacerbated harvest effects with decreasing trophic level, though these effects were largely attenuated by population size changes in the top predator. **Black** lines represent outcomes with evolutionary and ecological processes included; **red** lines represent outcomes with evolution frozen and only ecological processes following the initiation of harvest. Evolution in odd-numbered trophic levels increased harvested species yield and stability, while evolution in even-numbered trophic levels decreased yield and stability. Lines represent mean ± one standard deviation for twelve runs per each point.

Figure S7.

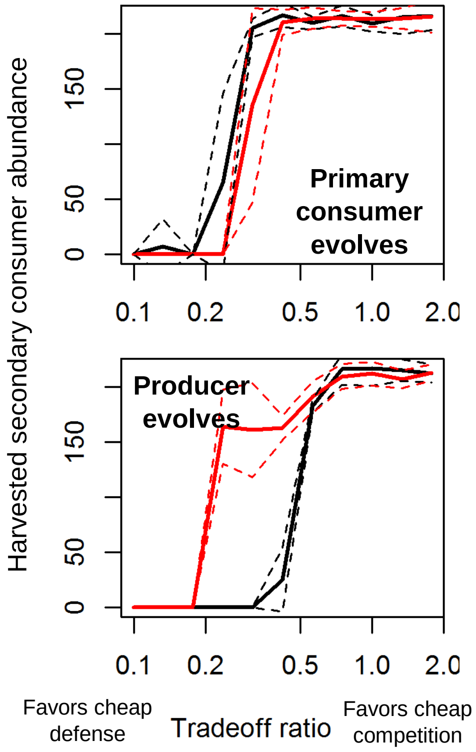

Competition-defense tradeoffs and eco-evolutionary potential. Secondary consumer (penultimate trophic level) harvested. Intermediate competition-defense tradeoff ratios in non-harvested species led to divergence between eco-evolutionary (**Black**) and ecology-only (**Red**) model outcomes for harvested species abundance. Tradeoff ratios necessary to cause significant evolution in response to harvest became more biased towards inexpensive defense as trophic level increased. Tradeoffs biased strongly towards inexpensive defense led to food web collapse and extirpation of the harvested species; tradeoffs biased towards inexpensive competition caused no difference between eco-evolutionary and ecology-only models. Lines represent mean ± one standard deviation for twelve runs per each point.
